# The New Orleans Medical and Surgical Journal

**Published:** 1873-10-01

**Authors:** 


THE NEW ORLEANS MEDICAL AND SURGICAL JOURNAL.—We have received the first and second number of the new series of this very valuable medical periodical. It is published bi-monthly, under the editorial supervision of Dr. S. M. Bemiss, and we heartily recommend it to all who desire a medical journal from the Southwest.

